# Intergenerational hypertension transmission before and after South Africa's democratic transition

**DOI:** 10.1016/j.ssmph.2026.101908

**Published:** 2026-03-15

**Authors:** Winfred A. Avogo

**Affiliations:** Department of Sociology and Anthropology, Illinois State University, Normal, IL, 61790, USA

**Keywords:** Hypertension, Intergenerational health transmission, Weathering hypothesis, Structural racism, Life course epidemiology, Post-apartheid South Africa, Natural experiment

## Abstract

**Background:**

Apartheid's structural violence produced profound cardiovascular inequalities among Black South Africans through processes that accelerate biological aging ("weathering") and transmit health disadvantage across successive generations. Whether South Africa's 1994 political transition interrupted this intergenerational transmission remains unknown. This study tests whether parent-to-offspring hypertension transmission weakened among cohorts born after apartheid's end, using the 1994 transition as a natural experiment.

**Methods:**

This study analyzed 30,438 parent-offspring dyads from five waves (2008–2017) of South Africa's National Income Dynamics Study, representing 11,655 unique offspring aged 18 years and older. Hypertension was defined as blood pressure ≥140/90mmHg or use of antihypertensive medication. Logistic regression models with offspring-clustered standard errors estimated transmission strength by birth cohort (born before 1960; born 1960–1993; born 1994 or later), testing whether socioeconomic factors (education, income, province) attenuated parent-offspring associations.

**Results:**

Parent-offspring hypertension transmission remained strong after adjusting for demographics and socioeconomic factors (OR = 1.83, 95% CI: 1.71–1.95). Transmission strength was statistically invariant across birth cohorts, with no significant weakening among those born after 1994 (OR = 1.63) compared with those born during the apartheid era (OR = 1.83). Socioeconomic controls produced minimal attenuation (a 7% reduction), suggesting that biological embedding predominates over structural persistence. Cohort-invariant transmission persisted among Black Africans (OR = 1.77) and Coloureds (OR = 2.05), with birth cohort interactions failing to reach statistical significance in both groups.

**Conclusions:**

Political liberation dismantled apartheid's legal structures but failed to interrupt intergenerational cardiovascular transmission. The "Born Free" generation inherits hypertension vulnerability at rates indistinguishable from apartheid-era cohorts despite improved socioeconomic conditions. This persistence suggests that biological embedding mechanisms transcend political change, requiring interventions that target both structural conditions and biological pathways.

## Highlights

• We examine hypertension transmission across 30,443 parent-offspring dyads in South Africa.
• Parent.→offspring HTN transmission shows no weakening across apartheid-era birth cohorts
• Structural health inequalities persist despite South Africa's 1994 democratic transition.
• Apartheid-era residential segregation continues to shape cardiovascular health patterns.
• Political reforms alone insufficient to interrupt intergenerational health inequality.

## 1 Introduction

In post-apartheid South Africa, non-communicable diseases have surpassed infectious diseases as leading causes of mortality. Between 1997 and 2018, NCD-related deaths rose by 58.7%; by 2020, NCDs accounted for more deaths than all infectious diseases combined (Bradshaw et al., 2003; Statistics South Africa, 2021). Hypertension prevalence now exceeds 40% nationally, following a steep social gradient with mortality among the urban poor nearly double that of wealthier populations (Gómez-Olivé et al., 2017; Nojilana et al., 2016; Shisana et al., 2014).

These epidemiological patterns raise a fundamental question for health equity: if apartheid embedded cardiovascular risk in the parental generation through structural violence and chronic stress, can political liberation alone break the chain of transmission to their children? Democracy dismantled apartheid's legal architecture, but the cardiovascular consequences persist, are transmitted across generations, and reproduce as health inequity in a formally equal society. Whether transmission operates primarily through biological pathways — where parental weathering under apartheid created durable physiological vulnerability — or through structural pathways — where persistent spatial segregation, economic inequality, and healthcare gaps continue exposing both generations to similar risk environments — remains empirically uncertain. This distinction carries profound implications for what political liberation can and cannot accomplish, as well as for the design of interventions capable of actually interrupting intergenerational cardiovascular inequity.

### 1.1 Theoretical framework: biological embedding vs. structural persistence

#### 1.1.1 Weathering and the intergenerational question

The "weathering" hypothesis, first proposed by Geronimus (Geronimus, 1992), holds that marginalized populations experience early health deterioration as a physiological consequence of cumulative socioeconomic struggle and political exclusion. Chronic exposure to racism and the sustained high-effort coping required to navigate discriminatory environments produce profound physiological effects through allostatic load — the cumulative wear and tear on biological systems from chronic stress response activation (McEwen & Stellar, 1993). This weathering manifests in measurable biomarkers: elevated stress hormones, increased blood pressure, altered metabolic markers, and accelerated cellular aging (Seekings & Nattrass, 2005; Williams & Mohammed, 2013).

Empirical evidence confirms this biological reality. Black Americans exhibit accelerated telomere attrition with cellular aging markers equivalent to those of White Americans nearly a decade older (Geronimus et al., 2010). Black adults show significantly higher allostatic load scores than White adults across all age groups, with disparities widening in mid-life (Geronimus et al., 2006). Critically, these racial differences persist independent of poverty, suggesting that weathering reflects cumulative exposure to structural racism rather than contemporaneous economic deprivation alone (Geronimus et al., 2006). Apartheid's structural violence — passbook restrictions, forced removals, Bantu education, state-sanctioned discrimination — created precisely the chronic, inescapable stressor environment theorized to generate weathering effects among Black South Africans (Mujahid et al., 2008; World Bank, 2022).

While weathering explains how structural violence becomes biologically embedded in individuals across the life course, a critical extension remains underexplored: does this weathered physiological state transfer across generations? If apartheid's cumulative stress damaged parental cardiovascular systems, does that damage reset at birth, or is it transmitted to offspring through intrauterine environments, epigenetic modifications, or shared risk exposures? This intergenerational question serves as the theoretical framework for the present study.

#### 1.1.2 Two competing mechanisms of intergenerational transmission

The conceptual framework is centered on the tension between biological embedding and structural persistence. These mechanisms are not mutually exclusive; rather, they represent distinct temporalities and pathways through which apartheid's legacy manifests. Both predict strong parent-offspring correlation in hypertension, but they differ critically in their predictions about whether — and how — political liberation should interrupt transmission.

*Biological Embedding: The Weathering Pathway.* This mechanism holds that apartheid's physiological damage became locked into parental biology before liberation, creating durable transmission pathways that persist despite offspring's current circumstances. Once established in parental physiology, weathering creates high-risk developmental environments for offspring through multiple biological pathways: epigenetic programming, whereby parental stress alters DNA methylation patterns in germ cells, transmitting vulnerability independent of offspring's environmental exposures (Barker, 1995; Gluckman et al., 2008); in-utero exposures, whereby maternal hypertension and chronic stress elevate circulating cortisol that crosses the placental barrier, programming fetal cardiovascular development along the lines of what Barker termed the "developmental origins of health and disease" (Barker, 1995); and the biological inheritance of weathering itself, including telomere shortening, accelerated cellular aging, and persistent inflammatory activation that create heritable physiological vulnerability (Chae et al., 2014).

If biological embedding dominates, offspring hypertension represents a biological echo of parental struggle under apartheid. The parent-offspring correlation persists even when offspring achieve educational mobility, residential desegregation, or improved economic circumstances, because risk was embedded during critical developmental windows — in parental gametogenesis, fetal programming, or early childhood — before liberation occurred. Critically, this mechanism predicts that political transition alone cannot interrupt transmission, since the biological damage occurred before regime change.

*Structural Persistence: The Architecture Pathway.* Alternatively, structural persistence holds that parent-offspring correlation reflects intergenerational reproduction of cardiovascular risk environments rather than biological inheritance. The correlation represents shared contemporary exposure to social determinants of health rather than inherited embodiment (Mujahid et al., 2008). Apartheid's spatial and economic architecture — designed to concentrate Black South Africans in under-resourced townships — did not dissolve with political liberation (Chae et al., 2014; Chatterjee et al., 2022).

Spatial persistence operates through multiple pathways. Although apartheid's legal framework was dismantled in 1994, its spatial legacy remains profound: the Group Areas Act's residential segregation persists informally, with Black families concentrated in disadvantaged township environments their parents inhabited (Chatterjee et al., 2022; StataCorp. Stata Statistical Software, 2021). These neighborhoods function as cardiovascular risk environments through chronic violence generating sustained stress, limited fresh food access, constrained physical activity, and environmental exposures including air pollution (Harris et al., 2011; Nowers et al., 2022; World Bank, 2022). Economic reproduction perpetuates disadvantage through South Africa's extraordinary inequality — with a Gini coefficient among the highest globally — reflecting profound wealth gaps rooted in apartheid's systematic asset stripping (Leibbrandt et al., 2010). Healthcare access barriers further entrench familial clustering: apartheid's two-tier health system created infrastructure inequalities that persisted after 1994, leaving Black families disproportionately reliant on overburdened public facilities while the private sector serves predominantly wealthier populations (Coovadia et al., 2009; Southern Africa Labour and Development Research Unit (SALDRU), 2018). In this model, the offspring's body reacts to ongoing structural violence — the same environmental triggers that caused parental hypertension — rather than to inherited biology.

#### 1.1.3 Distinguishing the mechanisms: the 1994 transition as an empirical test

South Africa's 1994 political transition provides unique quasi-experimental leverage to adjudicate between these mechanisms. If post-apartheid structural reforms meaningfully altered cardiovascular risk environments, parent-offspring hypertension associations should weaken for cohorts born after liberation — offspring who experienced less direct apartheid exposure and theoretically gained greater access to educational opportunities, residential mobility, and healthcare services. Conversely, if biological embedding dominates, parent-offspring associations should remain cohort-invariant, with uniform transmission strength across offspring birth years. Parental weathering occurred before liberation; biological damage was already locked in before regime change, and offspring born afterward inherit this vulnerability through pathways that transcend contemporary structural conditions.

This quasi-experimental logic depends critically on whether a "liberation dividend" proved real — whether post-1994 structural improvements were sufficient to alter cardiovascular risk environments. Evidence suggests limited progress: residential segregation persisted informally, wealth inequality increased, and healthcare access gaps remained substantial (Southern Africa Labour and Development Research Unit (SALDRU), 2018; StataCorp. Stata Statistical Software, 2021; Chatterjee et al., 2022). This creates interpretive ambiguity: cohort-invariant transmission does not definitively prove biological embedding dominates, since it could equally emerge from structural conditions that improved only marginally. What cohort stratification reveals is whether political transition coincided with a change in transmission strength — a necessary condition for structural persistence to weaken — and whether socioeconomic controls attenuate parent-offspring associations, as structural persistence would predict.

#### 1.1.4 Study contribution

This study presents the first nationally representative test of intergenerational cardiovascular transmission in South Africa. Using five waves (2008–2017) of the National Income Dynamics Study, the analysis utilizes parent-offspring dyads spanning three decades of birth cohorts and compare transmission strength among offspring born under apartheid versus those born after liberation. We examine whether socioeconomic controls — education, household income, province — attenuate parent-offspring associations, which would support structural persistence, or whether minimal attenuation occurs, suggesting biological embedding predominates. This analysis further test whether transmission patterns differ across apartheid-era population groups — Black African and Coloured — whose distinct exposures to structural violence may produce different intergenerational signatures. This is the first study using nationally representative data to test whether political liberation interrupted the intergenerational transmission of health inequity inscribed in the preceding generation.

## 2 Methods

### 2.1 Data source and study design

This study draws data from the National Income Dynamics Study (NIDS), South Africa's first nationally representative longitudinal household panel study. NIDS tracks individuals and households over time regardless of migration, residential mobility, or household dissolution—a critical design feature for intergenerational research in contexts of high spatial mobility. The study was launched in 2008 by the Southern Africa Labour and Development Research Unit (SALDRU) at the University of Cape Town, with subsequent waves collected in 2010–2011 (Wave 2), 2012 (Wave 3), 2014–2015 (Wave 4), and 2017 (Wave 5). NIDS employs a stratified, two-stage cluster sample design to achieve national representativeness across South Africa's nine provinces and diverse population groups.

NIDS has been extensively used to examine health inequalities in post-apartheid South Africa, establishing it as a reliable data source for studying population health dynamics. Prior research using NIDS has documented inequities in non-communicable disease multimorbidity patterns (Roomaney et al., 2022), spatial and temporal variation in mental health outcomes (Tomita et al., 2019), relationships between income inequality and depressive symptoms (Adjaye-Gbewonyo et al., 2016), and health disparities among inter-provincial migrants (Akinyemi et al., 2021). This body of research demonstrates NIDS's capacity to capture complex health inequalities and intergenerational transmission processes in South Africa's post-transition context.

The longitudinal panel structure provides unique advantages for studying intergenerational health transmission. Unlike cross-sectional surveys capturing parent-offspring relationships only when co-resident, NIDS maintains kinship linkages even when adult children establish independent households. The household roster records biological mother and father identifiers, enabling reconstruction of parent-offspring dyads across residential contexts. This design is particularly suited to South Africa's quasi-experimental setting: offspring birth cohorts spanning the 1994 apartheid transition can be directly compared on intergenerational hypertension transmission.

### 2.2 Sample construction

Parent-offspring dyads were constructed to facilitate the intergenerational analysis by linking biological parents to their adult children (aged 18 years and above) using kinship identifiers recorded in household rosters across all five NIDS waves. When both parents participated with valid blood pressure data, offspring contributed both a mother-offspring and father-offspring observation; when only one parent had valid data, offspring contributed a single dyad. Blood pressure measurements **were merged from** the Adult Health questionnaire for both generations, retaining only dyads where both had valid hypertension assessments in the same wave.

### 2.3 Measures

#### 2.3.1 Hypertension assessment

Hypertension was assessed using measured blood pressure from the Adult Health questionnaire administered in each NIDS wave. Trained fieldworkers measured systolic and diastolic blood pressure twice on the participant's left arm after at least 5 min of seated rest, using automated digital oscillometric devices. We calculated mean systolic blood pressure (SBP) and mean diastolic blood pressure (DBP) by averaging the two measurements. Hypertension was defined as mean SBP of 140 mmHg or above, OR mean DBP of 90 mmHg or above, OR current use of antihypertensive medication, consistent with the standard threshold used in South African population health research (Gómez-Olivé et al., 2017; Shisana et al., 2014) and aligned with established clinical guidelines (Whelton et al., 2017).

Biologically implausible values were excluded (SBP below 60 or above 260 mmHg; DBP below 30 or above 160 mmHg), removing fewer than 0.5% of observations.

Hypertension assessment protocols remained consistent across NIDS waves, permitting pooling of observations without introducing measurement non-equivalence.

#### 2.3.2 Birth cohort stratification

This study categorizes offspring into three birth cohorts based on estimated year of birth: (1) born before 1960 (pre-apartheid peak), (2) born 1960–1993 (apartheid era), and (3) born 1994 or later (post-apartheid/"Born Free" generation). Birth year was calculated by subtracting offspring's age at interview from interview year. These boundaries reflect critical junctures: 1960 marks the Sharpeville Massacre and intensification of apartheid violence; 1994 marks democratic transition and formal dismantling of apartheid's legal architecture.

The 1960 lower boundary reflects a data constraint rather than theoretical choice. Because NIDS launched in 2008, individuals born before 1948 would be 60 years or older at first measurement and thus occupy the parent rather than offspring generation in our intergenerational design. The 1960 cutoff ensures the pre-apartheid peak cohort remains analytically distinct while maintaining sufficient sample size. The concentration of observations in the 1960–1993 apartheid-era cohort (82.3%) provides substantial statistical power for detecting transmission effects, while the cohort born 1994 or later (16.3%) permits reliable testing of whether democratic reforms interrupted intergenerational transmission.

The 1994 threshold provides quasi-experimental leverage for distinguishing biological embedding from structural persistence mechanisms. Offspring born after 1994 theoretically experienced less direct exposure to apartheid's structural violence and greater access to residential mobility, educational opportunity, and healthcare services under democracy. If structural conditions improved post-liberation, structural persistence predicts weakening parent-offspring hypertension transmission in the cohort born 1994 or later. Conversely, if parental weathering locked cardiovascular vulnerability into biology before 1994, transmission should remain cohort-invariant.

#### 2.3.3 Population group classification

Following NIDS protocols, apartheid-era population group classification was used— African, Coloured, Indian/Asian, White — as proxy for differential exposure to apartheid's structural violence. These categories, though politically constructed rather than biologically meaningful, capture gradations in cumulative exposure to residential segregation, educational exclusion, economic exploitation, and political violence. Black Africans experienced the most severe structural racism; Coloureds faced intermediate discrimination; Whites were largely exempt.

This classification is retained for two reasons. First, it permits testing whether intergenerational transmission strength differs by historical exposure intensity. Second, it acknowledges that post-1994 deracialization has not eliminated the material consequences of apartheid's racial stratification — residential segregation, wealth inequality, and healthcare access gaps remain strongly patterned by apartheid-era classification. The analysis examines whether transmission mechanisms differ by historical exposure intensity, not whether population groups are biologically distinct.

#### 2.3.4 Socioeconomic covariates

To assess whether socioeconomic factors mediate intergenerational hypertension transmission, this study utilizes offspring education level (categorized as no schooling, primary, incomplete secondary, secondary/matric, or post-secondary), household income per capita (log-transformed to address skewness), and province of residence (South Africa's nine provinces as fixed effects). These controls permit testing whether parent-offspring hypertension associations reflect primarily structural persistence (transmission operating through accumulated disadvantage) or biological embedding (transmission operating through maternal weathering and epigenetic mechanisms persisting despite improved material conditions).

The analysis also controlled for offspring characteristics that could confound the parent-offspring association: age (continuous years), gender (male/female), and employment status (employed vs. not employed). These covariates isolate the intergenerational component of hypertension risk from offspring's own contemporaneous socioeconomic and demographic determinants.

#### 2.3.5 Statistical analysis

This study estimated parent-offspring hypertension transmission strength using logistic regression with offspring hypertension status (yes/no) as dependent variable and parent hypertension status (yes/no) as primary independent variable. The odds ratio on parent hypertension quantifies intergenerational transmission.

The analytical strategy proceeded in three stages. Model 1 examined the unadjusted association between parent and offspring hypertension, including offspring birth cohort and the parent hypertension by cohort interaction. This tests whether transmission strength differs across cohorts born before 1960, during apartheid (1960–1993, reference), and after democratic transition (1994 or later). A significant interaction would indicate transmission weakened or strengthened for specific cohorts, supporting structural persistence if weakening occurred after 1994 or biological embedding if transmission remained cohort-invariant. Model 2 added demographic covariates: offspring age, gender, and employment. Model 3 incorporated socioeconomic controls (education, household income, province) to assess whether these factors attenuate the parent-offspring association, supporting structural persistence mechanisms. Interaction significance was assessed using Wald tests.

Population group heterogeneity: To examine whether transmission patterns differ by historical apartheid exposure, we estimated Model 3 separately for Black African and Coloured subsamples (Table 3). Indian/Asian and White subsamples were too small for reliable stratified analysis (n = 377 and n = 522, respectively). These models test whether groups experiencing more severe apartheid exposure exhibit stronger parent-offspring associations and whether cohort patterns differ across groups.

Age measurement and interpretation: The generational age gap between parents (typically middle-aged or older) and offspring (predominantly young adults) produces expected baseline differences in hypertension prevalence, as cardiovascular risk accumulates with age. We control for offspring age continuously in Models 2 and 3 to ensure intergenerational associations reflect transmission mechanisms rather than age-related risk accumulation. Our key parameter — the odds ratio comparing offspring of hypertensive versus normotensive parents — isolates the intergenerational component after adjusting for offspring age. This study acknowledges that the cohort born in 1994 or later is relatively young (maximum age approximately 23 at Wave 5 in 2017), and some individuals may develop hypertension later. However, this measurement timing concern would bias estimates toward the null (weakening observed transmission in younger cohorts), making the cohort-invariance findings conservative.

Standard error adjustment: Because offspring measured across multiple waves and those with both parents measured contribute multiple observations, observations are not statistically independent. Robust standard errors were computed and clustered at the offspring level in all models, ensuring valid statistical inference by allowing arbitrary correlation among observations for the same offspring while maintaining independence assumptions across different offspring.

All analyses were conducted in Stata 17 (StataCorp. Stata Statistical Software, 2021). Statistical significance was assessed at the 0.05 level (two-tailed).

#### 2.3.6 Ethical approval

NIDS received ethical clearance from the University of Cape Town's Commerce Faculty Ethics Committee. All participants provided written informed consent. Our secondary analysis of de-identified NIDS data was deemed exempt from further review by the Illinois State University Institutional Review Board.

## 3 Results

### 3.1 Sample characteristics

The analytic sample comprises 30,438 parent-offspring dyad observations representing 11,655 unique offspring measured across five NIDS waves (2008–2017). Table 1 presents complete sample characteristics. The sample includes 22,148 maternal dyads (72.8%) and 8290 paternal dyads (27.2%), reflecting higher female participation in NIDS health assessments and mothers' greater likelihood of co-residing with adult children. Parent hypertension prevalence was 71.3%, while offspring prevalence was substantially lower (40.8%), consistent with the generational age gap (parents mean = 56.0 years, SD = 10.9; offspring mean = 27.1 years, SD = 8.4).

**Table 1:** Sample characteristics of parent-offspring dyads (N = 30,438).

		
Characteristic	N (categorical)/Mean (continuous)	% (categorical)/SD (continuous)
**Parent Characteristics**		
Total parent-offspring dyads	30,438	
Unique offspring	11,655	
Hypertension prevalence	21,717	71.3
Maternal dyads	22,148	72.8
Paternal dyads	8290	27.2
Age, years	56.0	10.9
**Offspring Characteristics**		
Hypertension prevalence	12,433	40.8
Age, years	27.1	8.4
Male	13,903	45.7
Female	16,535	54.3
Birth cohort		
Born before 1960	423	1.4
Born 1960–1993 (Apartheid era)	25,047	82.3
Born 1994 or later (Post-apartheid)	4968	16.3
Population group		
Black African	25,060	82.3
Coloured	4467	14.7
Indian/Asian	377	1.2
White	522	1.7
Currently employed	7562	24.8
**Socioeconomic Characteristics**		
Education		
No schooling	599	2.0
Primary (Grades 1–7)	3158	10.4
Secondary incomplete (Grades 8–11)	14,592	47.9
Matric/Grade 12	7203	23.7
Post-secondary	4886	16.1
Household income per capita (Rand/month)		
Mean	1464.6	3229.0
Median	731.6	
Province		
Western Cape	3222	10.6
Eastern Cape	3237	10.6
Northern Cape	1997	6.6
Free State	1653	5.4
KwaZulu-Natal	9089	29.9
North West	2631	8.6
Gauteng	3032	10.0
Mpumalanga	2042	6.7
Limpopo	3535	11.6

NIDS Waves 1–5 (2008–2017).

HTN = Hypertension (measured blood pressure ≥140/90 mmHg or taking antihypertensive medication).

For categorical variables, columns present N (sample size) and % (percentage); for continuous variables, columns present Mean and SD (standard deviation).

Sample restricted to parent-offspring dyads with complete data on all covariates.

The birth cohort distribution reflects South Africa's demographic history: 423 offspring born before 1960 (1.4%), 25,047 born during apartheid 1960–1993 (82.3%), and 4968 born after liberation in 1994 or later (16.3%). The sample is predominantly Black African (82.3%), followed by Coloured (14.7%), with small Indian/Asian (1.2%) and White (1.7%) subsamples. Regarding socioeconomic characteristics, nearly half of the offspring (47.9%) had incomplete secondary education, while 23.7% completed matric and 16.1% attained post-secondary education. Median household income per capita was 732 Rand per month, and only 24.8% of offspring were currently employed.

### 3.2 Intergenerational hypertension transmission: model progression

Table 2 presents logistic regression results estimating parent-to-offspring hypertension transmission across three model specifications. Model 1 includes only parent hypertension status, offspring birth cohort, and their interaction, unadjusted for covariates. Parents with hypertension had 1.91 times higher odds of having hypertensive offspring compared to normotensive parents (95% CI: 1.79–2.04, p < 0.001). The interaction between parent hypertension and offspring cohort was not statistically significant (χ^2^ = 1.13, p = 0.57), providing initial evidence of cohort-invariant transmission.

**Table 2:** Logistic regression models: Intergenerational hypertension transmission.

	Model 1	Model 2	Model 3
**Main Effects**			
Parent hypertension (vs. no hypertension)	1.908⨯⨯⨯	1.902⨯⨯⨯	1.825⨯⨯⨯
	*(0.061)*	*(0.062)*	*(0.060)*
Born before 1960 (vs. born 1960–1993)	3.004⨯⨯⨯	0.938	0.932
	*(0.786)*	*(0.268)*	*(0.266)*
Born 1994 or later (vs. born 1960–1993)	0.483⨯⨯⨯	0.682⨯⨯⨯	0.672⨯⨯⨯
	*(0.035)*	*(0.051)*	*(0.051)*
**Cohort Interactions (Parent Hypertension × Birth Cohort)**			
Parent hypertension × Born before 1960	0.932	0.932	0.879
	*(0.258)*	*(0.272)*	*(0.253)*
Parent hypertension × Born 1994 or later	0.893	0.900	0.891
	*(0.073)*	*(0.074)*	*(0.074)*
**Demographics**			
Age (years)	—	1.042⨯⨯⨯	1.041⨯⨯⨯
		*(0.002)*	*(0.003)*
Female (vs. male)	—	0.660⨯⨯⨯	0.644⨯⨯⨯
		*(0.022)*	*(0.022)*
Employed (vs. not employed)	—	1.091⨯	0.993
		*(0.040)*	*(0.039)*
**Socioeconomic Status (reference: Matric/Grade 12)**			
No schooling	—	—	1.154
			*(0.145)*
Primary education	—	—	1.110
			*(0.069)*
Incomplete secondary	—	—	0.924
			*(0.038)*
Post-secondary	—	—	0.999
			*(0.053)*
Log(income per capita +1)	—	—	0.997
			*(0.018)*
**Province (reference: Western Cape)**			
Eastern Cape	—	—	0.583⨯⨯⨯
			*(0.043)*
Northern Cape	—	—	0.740⨯⨯⨯
			*(0.062)*
Free State	—	—	0.589⨯⨯⨯
			*(0.051)*
KwaZulu-Natal	—	—	0.540⨯⨯⨯
			*(0.033)*
North West	—	—	0.582⨯⨯⨯
			*(0.045)*
Gauteng	—	—	0.501⨯⨯⨯
			*(0.038)*
Mpumalanga	—	—	0.373⨯⨯⨯
			*(0.032)*
Limpopo	—	—	0.419⨯⨯⨯
			*(0.031)*
**Model Fit**			
Observations	30,438	30,438	30,434
Log likelihood	−19,914.67	−19,473.31	−19,224.27
Chi-square	899.67	1317.47	1524.17

Coefficients are odds ratios. Standard errors (clustered by offspring) in parentheses.

Model 1: Parent hypertension and birth cohort interaction only (N = 30,438). Model 2: Model 1 plus offspring age, gender, and employment status (N = 30,438). Model 3: Model 2 plus offspring education, log household income per capita, and province fixed effects (N = 30,434); four observations excluded due to sparse cells within the province × education × cohort design matrix, consistent with complete separation in logistic regression. Excluding these observations does not affect substantive conclusions. Reference categories: Born 1960–1993 (birth cohort); no hypertension (parent hypertension); male (gender); not employed (employment); Matric/Grade 12 (education); Western Cape (province).

— = not included in model.

⨯ p < 0.05, ⨯⨯ p < 0.01, ⨯⨯⨯ p < 0.001.

**Table 3:** Intergenerational hypertension transmission by population group (Model 3 with socioeconomic controls).

	Black African	Coloured
**Main Effects**		
Parent hypertension (vs. no hypertension)	1.772⨯⨯⨯	2.055⨯⨯⨯
	*(0.064)*	*(0.175)*
Born before 1960 (vs. born 1960–1993)	0.811	2.380
	*(0.248)*	*(2.944)*
Born 1994 or later (vs. born 1960–1993)	0.682⨯⨯⨯	0.596⨯⨯
	*(0.057)*	*(0.119)*
**Cohort Interactions (Parent Hypertension × Birth Cohort)**		
Parent hypertension × Born before 1960	0.917	0.827
	*(0.282)*	*(1.082)*
Parent hypertension × Born 1994 or later	0.901	0.887
	*(0.083)*	*(0.184)*
**Demographics**		
Age (years)	1.043⨯⨯⨯	1.038⨯⨯⨯
	*(0.003)*	*(0.007)*
Female (vs. male)	0.630⨯⨯⨯	0.650⨯⨯⨯
	*(0.023)*	*(0.061)*
Employed (vs. not employed)	0.972	1.123
	*(0.043)*	*(0.098)*
**Socioeconomic Status (reference: Matric/Grade 12)**		
No schooling	1.197	0.825
	*(0.155)*	*(0.490)*
Primary education	1.136	0.943
	*(0.077)*	*(0.159)*
Incomplete secondary	0.963	0.806
	*(0.043)*	*(0.094)*
Post-secondary	1.054	0.813
	*(0.060)*	*(0.126)*
Log(income per capita +1)	0.976	0.914
	*(0.020)*	*(0.050)*
**Province (reference: Western Cape)**		
Eastern Cape	0.780⨯	0.764
	*(0.096)*	*(0.122)*
Northern Cape	0.829	0.772⨯
	*(0.129)*	*(0.080)*
Free State	0.848	0.381
	*(0.110)*	*(0.192)*
KwaZulu-Natal	0.752⨯	1.002
	*(0.086)*	*(0.256)*
North West	0.800	0.977
	*(0.099)*	*(0.828)*
Gauteng	0.683⨯⨯	0.679
	*(0.085)*	*(0.161)*
Mpumalanga	0.525⨯⨯⨯	n/a
	*(0.068)*	
Limpopo	0.577⨯⨯⨯	1.057
	*(0.070)*	*(0.783)*
**Model Fit**		
Observations	25,060	4467

Coefficients are odds ratios. Standard errors (clustered by offspring) in parentheses.

Model includes full socioeconomic controls: education, household income per capita, and province fixed effects.

Reference categories: Born 1960–1993 (birth cohort); no hypertension (parent hypertension); male (gender); not employed (employment); Matric/Grade 12 (education); Western Cape (province).

Cohort × parent hypertension interaction: Black African p = 0.519; Coloured p = 0.839.

n/a = insufficient observations in this province-group cell for reliable estimation.

Indian/Asian (n = 377) and White (n = 522) subsamples excluded due to insufficient sample size for reliable stratified analysis. 12 observations missing population group data not shown.

— = not applicable. ⨯ p < 0.05, ⨯⨯ p < 0.01, ⨯⨯⨯ p < 0.001.

Model 2 added demographic covariates — offspring age, gender, and employment status. The parent hypertension effect remained virtually unchanged (OR = 1.90, 95% CI: 1.78–2.03, p < 0.001), and the cohort interaction remained non-significant (χ^2^ = 2.08, p = 0.35). As expected, offspring hypertension risk increased with age (OR = 1.04 per year, 95% CI: 1.04–1.05, p < 0.001), and female offspring had a lower risk than male offspring (OR = 0.66, 95% CI: 0.62–0.70, p < 0.001). Employment status showed a modest positive association (OR = 1.09, 95% CI: 1.02–1.16, p = 0.016).

Model 3 incorporated socioeconomic controls — offspring education, household income per capita (log-transformed), and province fixed effects — to assess whether these factors mediate intergenerational transmission. The parent hypertension effect remained robust (OR = 1.83, 95% CI: 1.71–1.95, p < 0.001), representing only minimal attenuation from Model 2 (from 1.90 to 1.83, a 7% reduction in odds ratio). The cohort interaction remained non-significant (χ^2^ = 2.16, p = 0.34), confirming cohort-invariant transmission even after adjusting for contemporary socioeconomic conditions.

### 3.3 Socioeconomic factors and transmission mechanisms

The minimal attenuation after adding socioeconomic controls provides critical evidence regarding transmission mechanisms. Neither offspring education nor household income significantly predicted offspring hypertension risk in Model 3. Compared to offspring with matric (Grade 12) education, those with no schooling (OR = 1.15, 95% CI: 0.90–1.47, p = 0.255), primary education (OR = 1.11, 95% CI: 0.98–1.25, p = 0.095), incomplete secondary (OR = 0.92, 95% CI: 0.84–1.00, p = 0.054), or post-secondary education (OR = 1.00, 95% CI: 0.90–1.11, p = 0.990) showed no significant differences. Log household income per capita similarly exhibited no association with offspring hypertension (OR = 1.00, 95% CI: 0.97–1.04, p = 0.822).

In contrast, the province of residence exerted strong independent effects, with all provinces showing significantly lower hypertension risk than the Western Cape. The largest protective effects appeared in Mpumalanga (OR = 0.37, 95% CI: 0.32–0.43, p < 0.001) and Limpopo (OR = 0.42, 95% CI: 0.36–0.49, p < 0.001). These provincial differences likely reflect geographic variation in healthcare access, dietary patterns, and urbanization levels rather than structural persistence mechanisms, as the parent-offspring transmission strength remained consistent across provinces (evidenced by non-significant cohort interactions).

The persistence of strong parent-offspring associations despite socioeconomic adjustment (Model 2 OR = 1.90 vs. Model 3 OR = 1.83) supports biological embedding over structural persistence mechanisms. If structural disadvantage primarily mediated intergenerational transmission, we would expect substantial attenuation after controlling for offspring education and household income. Instead, the 7% reduction suggests that contemporary socioeconomic status explains minimal variance in offspring hypertension risk once parental hypertension status is accounted for. This pattern implies that maternal weathering during gestation and early life inscribes cardiovascular vulnerability into offspring biology in ways that persist independent of post-birth socioeconomic trajectories.

### 3.4 Cohort stratification: testing political transition effects

**Fig. 1:**
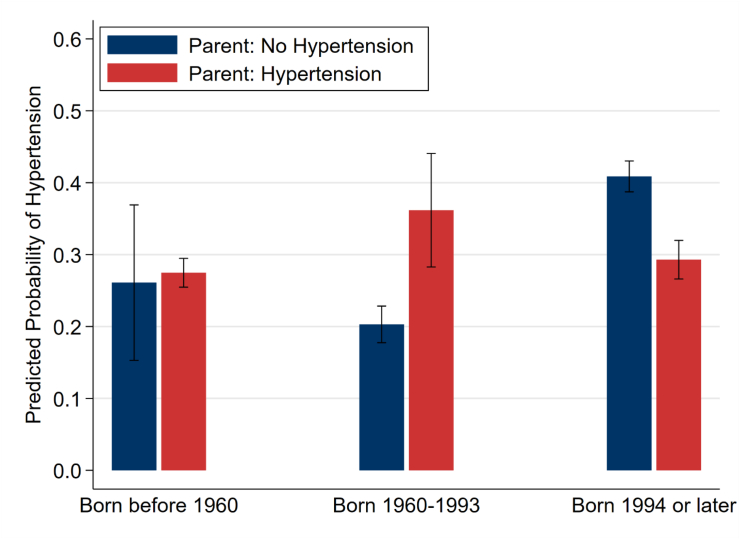
Predicted probability of offspring hypertension by parent hypertension status and birth cohort (Model 3 adjusted predictions). Error bars show 95% CIs. Consistent gap across cohorts demonstrates cohort-invariant transmission (interaction p = 0.34).

**Fig. 2:**
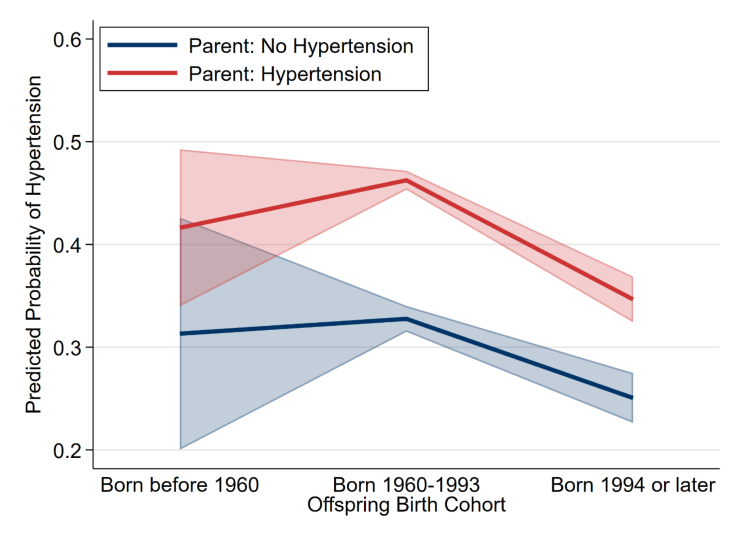
Predictive margins of parent hypertension status across offspring birth cohorts with 95% CIs (Model 3 adjusted predictions). Parallel lines demonstrate cohort-invariant intergenerational transmission (interaction p = 0.34).

To examine whether transmission strength differs across South Africa's democratic transition, we tested the interaction between parental hypertension and offspring cohort in all three models. The interaction was non-significant in Models 1 (p = 0.57), 2 (p = 0.35), and 3 (p = 0.34), indicating cohort-invariant transmission. Fig. 1, Fig. 2 visualize this pattern.

Examining cohort-specific odds ratios from Model 3, we find modest variation around a consistent transmission effect. For offspring born before 1960, the parent hypertension effect was OR = 1.61 (95% CI: 0.92–2.82, p = 0.094), though this estimate was imprecise due to small sample size (n = 423). For the reference cohort born 1960–1993, parent hypertension conferred an OR of 1.83 (95% CI: 1.72–1.95, p < 0.001). For offspring born 1994 or later, the effect was OR = 1.63 (95% CI: 1.40–1.89, p < 0.001). While point estimates varied slightly, confidence intervals overlapped substantially, and the formal interaction test confirmed no significant differences. The consistency of transmission strength across the 1994 democratic threshold provides strong evidence for biological embedding over structural persistence mechanisms.

Fig. 1 displays predicted probabilities of offspring hypertension by parent hypertension status and birth cohort, holding covariates at typical values (mean age, female, not employed, matric education, median income, KwaZulu-Natal province). Among offspring of normotensive parents, hypertension prevalence declined across successive cohorts (born before 1960: 26%; born 1960–1993: 27%; born 1994 or later: 20%), likely reflecting younger ages in the post-apartheid cohort. Critically, among offspring of hypertensive parents, prevalence followed a parallel trajectory (born before 1960: 36%; born 1960–1993: 41%; born 1994 or later: 29%), such that the gap between children of hypertensive versus normotensive parents remained constant across cohorts. This parallel pattern — rather than converging lines — provides visual confirmation of cohort-invariant transmission.

Fig. 2 presents the same pattern as an interaction plot, showing predicted probabilities for offspring with and without parental hypertension across cohorts. The two lines descend in parallel with overlapping confidence intervals, demonstrating that democratic reforms did not interrupt intergenerational transmission. The persistent vertical distance between lines indicates that parental hypertension confers similar excess risk regardless of whether offspring were born during apartheid, at the transition, or in the democratic era.

### 3.5 Population group heterogeneity

Table 3 examines whether intergenerational transmission patterns differ by apartheid-era population group classification, which proxies for differential exposure to structural violence. We estimated Model 3 separately for Black African and Coloured subsamples (Indian/Asian and White samples were too small for reliable stratified analysis).

Among Black Africans (n = 25,060), who experienced apartheid's most severe residential segregation and economic exploitation, parent-offspring hypertension transmission operated with similar strength to the overall sample (OR = 1.77, 95% CI: 1.65–1.90, p < 0.001). The cohort-by-parent hypertension interaction remained non-significant (χ^2^ = 1.31, p = 0.52), indicating invariant transmission across the 1994 threshold within the population group theoretically most likely to benefit from liberation policies. Neither education (all categories p > 0.05) nor log income (p = 0.227) significantly predicted offspring hypertension among Black Africans, reinforcing the minimal role of contemporary socioeconomic status in mediating transmission.

Among Coloureds (n = 4467), who faced intermediate apartheid exposure, transmission appeared numerically stronger (OR = 2.05, 95% CI: 1.76–2.40, p < 0.001), though confidence intervals overlapped with the Black African estimate. More importantly, the cohort interaction was non-significant (χ^2^ = 0.35, p = 0.84), demonstrating cohort-invariant transmission among Coloureds as well. Education and income similarly showed no significant associations with offspring hypertension in this subsample.

The consistency of cohort-invariant transmission across both Black Africans and Coloureds — who together constitute 97% of our sample and experienced the vast majority of apartheid's structural violence — demonstrates that the pattern is robust across differential historical exposure contexts. Whether groups faced severe or intermediate structural violence, the political transition did not interrupt intergenerational cardiovascular transmission. This cross-group consistency further supports biological embedding mechanisms, as structural persistence interpretations would predict differential cohort patterns depending on the magnitude of post-1994 material improvements, which theoretically varied across population groups.

### 3.6 Summary of key findings

Three primary findings emerge. First, strong parent-offspring hypertension transmission is observed across the sample (Model 3: OR = 1.83, 95% CI: 1.71–1.95, p < 0.001), with offspring of hypertensive parents having 83% higher odds of hypertension after adjusting for demographics and socioeconomic factors. Second, this transmission shows statistically invariant strength across birth cohorts spanning apartheid's final decades through the democratic era (cohort interaction p = 0.34), with no significant weakening among offspring born after liberation in 1994 or later. Third, minimal attenuation after adding socioeconomic controls (7% reduction from OR = 1.90 to OR = 1.83) and cohort-invariant patterns across both Black African and Coloured populations suggest that biological embedding mechanisms predominate over structural persistence pathways. Contemporary socioeconomic status — education, income, province — does not substantially explain offspring hypertension risk once parental hypertension status is accounted for, indicating that maternal weathering and early-life programming transmit cardiovascular vulnerability through biological channels that persist despite improved material conditions in the democratic era.

## 4 Discussion

This study tested whether South Africa's 1994 political transition interrupted intergenerational cardiovascular transmission. Three findings emerged: strong parent-offspring transmission persisted (OR = 1.83, 95% CI: 1.71–1.95) with statistically invariant strength across birth cohorts (interaction p = 0.34), minimal socioeconomic attenuation (7% reduction), and cohort-invariant patterns across both Black African and Coloured populations. Political transition alone did not interrupt intergenerational cardiovascular transmission in the first generation born under democracy.

### 4.1 Interpreting Cohort-Invariant Transmission: biological embedding

One interpretation invokes biological embedding through which parental weathering created physiological damage transmitted to offspring independent of contemporary conditions (Geronimus, 1992; Geronimus et al., 2006; Krieger, 2005; Seekings & Nattrass, 2005). Parents who lived through apartheid's peak decades experienced chronic stress, segregation, and deprivation that accelerated biological aging, manifesting as elevated allostatic load, shortened telomeres, and dysregulated cardiovascular function (Roomaney et al., 2022; Seekings & Nattrass, 2005; Williams & Mohammed, 2013). If this damage becomes biologically embedded through epigenetic modifications, in-utero programming, or inherited inflammatory profiles (Barker, 1995; Gluckman et al., 2008; Kim et al., 2023), it transmits regardless of whether offspring are born during or after apartheid.

The "Born Free" generation, theoretically unburdened by apartheid's direct restrictions, nonetheless may inherit their parents' weathered physiology (Kim et al., 2023). Even if these offspring grow up in desegregated neighborhoods and attend integrated schools, they carry forward cardiovascular vulnerability programmed during their parents' reproductive years or transmitted through maternal stress during pregnancy. The cohort-invariant pattern would thus reflect apartheid's violence becoming literally embodied in parents and passed through mechanisms transcending political structures (Krieger, 2005).

The findings that neither education nor income significantly predicts offspring hypertension (after accounting for parental status) provide tentative support for biological embedding. If transmission operated primarily through inherited socioeconomic disadvantage, we would expect substantial attenuation when controlling for these factors. Instead, the minimal 7% attenuation suggests parental hypertension transmits cardiovascular risk through channels relatively independent of offspring's achieved socioeconomic status, consistent with maternal weathering and early-life programming (Barker, 1995; Gluckman et al., 2008; Kim et al., 2023).

However, **this study cannot definitively establish biological embedding** due to the absence of direct biomarkers — telomere length (Chae et al., 2014), allostatic load scores (McEwen & Stellar, 1993), epigenetic markers, and inflammatory cytokines — to directly observe biological damage. Nor can we rule out that apparent biological transmission reflects unmeasured structural disadvantage operating through pathways not captured by education and income controls. The biological embedding interpretation remains plausible but unproven.

### 4.2 Alternative interpretation: persistent structural violence

Alternatively, transmission may remain invariant because the structural conditions that generate cardiovascular risk have barely improved despite the political transition. While apartheid's legal framework ended in 1994, its material consequences — residential segregation, wealth concentration, educational disparities — proved remarkably persistent (Leibbrandt et al., 2010; Mujahid et al., 2008). As one reviewer noted, "meaningful structural change does not happen overnight, and while the government changed in 1994, the structural barriers to health did not suddenly disappear for Black South Africans."

Quantitative evidence suggests limited structural transformation. South Africa's Gini coefficient increased from 0.59 in 1993 to 0.63 by 2014 (Leibbrandt et al., 2010). Home ownership rates among Black Africans remain below 50%, with most residing in former township areas (Chatterjee et al., 2022). The healthcare system remains two-tiered, with private care concentrated among affluent populations while the majority rely on under-resourced public clinics (Coovadia et al., 2009; Southern Africa Labour and Development Research Unit (SALDRU), 2018). Wealth disparities by race have barely narrowed despite affirmative action (Mujahid et al., 2008; Piraino, 2015).

### 4.3 Post-1994 social policies and their limitations

The democratic government implemented significant welfare programs. The Child Support Grant (1998) provides monthly cash transfers reaching over 12 million children by 2017. The Old Age Pension was expanded to provide universal non-contributory pensions to South Africans aged 60 and older. The Reconstruction and Development Programme aimed to deliver housing, electrification, and infrastructure to Black townships, building over 2 million houses by 2010 (World Bank, 2022).

However, these programs proved insufficient to fundamentally alter the structural conditions that generate cardiovascular risk. Cash transfers remained modest relative to poverty lines — the Child Support Grant provided approximately $30 USD per month, barely 10% of median household income in our sample (Leibbrandt et al., 2010). RDP housing often reproduced apartheid's spatial patterns by locating subsidized housing on urban peripheries far from employment, perpetuating long commutes and social isolation (Geronimus et al., 2006; Turok, 2014). Healthcare reforms improved access but did not eliminate the quality gap, and chronic disease management remained under-resourced (Coovadia et al., 2009; Southern Africa Labour and Development Research Unit (SALDRU), 2018). Educational desegregation did not eliminate quality disparities (Seekings & Nattrass, 2005).

Most critically, these policies primarily affected offspring born after 1994 rather than parents who had already accumulated weathering damage during apartheid (Geronimus, 1992; Geronimus et al., 2006; Seekings & Nattrass, 2005). Even if social grants modestly improved material conditions for offspring born 1994 or later, their parents' cardiovascular health — shaped by decades of structural violence before 1994 — remained fixed. If maternal hypertension during pregnancy programs offspring vulnerability in utero (Barker, 1995; Gluckman et al., 2008; Kim et al., 2023), then post-1994 policies arriving after birth would be too late to interrupt transmission for the first "Born Free" generation.

Under the structural persistence interpretation, offspring born after 1994 face cardiovascular risk environments sufficiently similar to those their parents navigated, so that transmission patterns remain unchanged. If residential segregation persists — what geographers term apartheid geography's "stickiness" (Chatterjee et al., 2022; Turok, 2014) — then offspring born 1994 or later grow up in neighborhoods with similar environmental stressors, food environments, and chronic stress as those born 1960–1993 (Harris et al., 2011; World Bank, 2022). Transmission would appear cohort-invariant, not because biological damage is irreversibly programmed, but because the structural violence that generates that damage persists in only a moderately attenuated form.

This interpretation has testability: families that escaped township geography or achieved substantial upward mobility should exhibit weaker transmission. Future research with granular residential histories, wealth trajectories, and neighborhood quality measures could adjudicate between mechanisms more definitively. The finding that transmission remains invariant even among offspring born 1994 or later — theoretically best positioned to benefit — suggests either biological embedding, weak structural change, or both operating simultaneously.

### 4.4 Acknowledging hypothesis testing limitations

The study design has important limitations for distinguishing mechanisms, as reviewers noted. The cohort stratification approach provides temporal leverage but limited mechanistic resolution. The null finding is consistent with multiple interpretations: (1) biological embedding dominates, with parental weathering creating irreversible damage transmitted through epigenetic or gestational pathways (Barker, 1995; Gluckman et al., 2008; Kim et al., 2023); (2) structural conditions barely improved, with the 1994 transition not producing sufficient material transformation (Chatterjee et al., 2022; Leibbrandt et al., 2010); (3) insufficient time elapsed, with structural improvements requiring multiple generations to manifest; or (4) both mechanisms operate simultaneously, reinforcing each other (Kim et al., 2023; Krieger, 2005).

Definitively adjudicating requires: biomarkers of biological transmission (Roomaney et al., 2022; Williams & Mohammed, 2013); sibling fixed-effects designs separating family from neighborhood effects; detailed residential and wealth trajectories (Piraino, 2015); or natural experiments identifying families that escaped townships. Our data lacks these resources. The minimum 7% attenuation provides suggestive evidence in favor of biological embedding but cannot rule out unmeasured structural persistence.

Moreover, oppression of Black South Africans did not begin with apartheid's 1948 formalization (Seekings & Nattrass, 2005). As one reviewer noted, "the situation for Black South Africans must have been bad prior to Apartheid — the formalizing of oppression in 1948 certainly meant that informal systems of oppression and racism were in place long before 1948." Colonial violence, land dispossession, and racial discrimination characterized South African society for centuries before apartheid. Our cohort born before 1960 nonetheless experienced profound structural violence under colonial and early apartheid regimes.

Despite these limitations, this study makes valuable contributions by formally testing whether 1994 legal changes resulted in an observable difference in intergenerational transmission whether 1994 legal changes resulted in an observable difference in intergenerational transmission — a question of theoretical and policy importance, even if the answer (no observable difference) does not definitively resolve mechanisms. The null finding is substantively meaningful: whatever processes drive intergenerational cardiovascular transmission proved resilient to macro-political change, suggesting interrupting these pathways requires more than legal reform. Our inclusion of socioeconomic controls provides additional leverage, with minimal attenuation (7%), offering tentative support for biological pathways. The consistency across both Black African and Coloured populations further suggests that mechanisms operate relatively independently of the severity of historical disadvantage.

### 4.5 Comparison to prior research

**These findings extend** prior South African research Von Fintel and Richter (Von Fintel & Richter, 2019), analyzing the Birth to Twenty cohort (Richter et al., 2007), documented intergenerational transfer of height, BMI, and blood pressure but could not test whether transmission changed post-apartheid (all births 1990). Our multi-cohort approach finds no temporal variation, suggesting transmission mechanisms operate with remarkable stability across political ruptures.

Internationally, the findings parallel US research on weathering among Black Americans, where physiological deterioration from discrimination persists across generations despite Civil Rights reforms (Geronimus, 1992; Geronimus et al., 2006; Seekings & Nattrass, 2005). However, US research lacks South Africa's quasi-experimental leverage. Where US racial inequality evolved gradually without clear intervention moments, South Africa's 1994 transformation provides a clearer temporal demarcation for testing whether political change interrupts health transmission. The null finding offers sobering evidence that political liberation alone proves insufficient to break intergenerational health chains forged under structural violence.

### 4.6 Policy implications

The findings carry important implications for post-conflict health equity. First, political liberation appears insufficient to interrupt intergenerational health transmission without accompanying material transformation. Breaking these chains likely requires interventions targeting both structural conditions (housing, neighborhood investment, healthcare access, wealth redistribution) and biological pathways (maternal health services, early childhood nutrition, stress reduction for weathered parents) (Coovadia et al., 2009; Southern Africa Labour and Development Research Unit (SALDRU), 2018).

The social policies implemented post-1994 represent meaningful attempts at redress but proved insufficient (Leibbrandt et al., 2010). Future interventions should be scaled more ambitiously, targeted earlier (ideally pre-conception and during pregnancy), and sustained over multiple generations. Programs addressing maternal cardiovascular health before and during pregnancy may prove particularly critical if in-utero programming contributes to transmission (Barker, 1995; Gluckman et al., 2008; Kim et al., 2023).

Second, standard interventions focusing exclusively on individual risk factors may prove inadequate when cardiovascular disease emerges from intergenerational trauma and structural violence (Bradshaw et al., 2006; Mayosi et al., 2009; World Health Organization, 2022). Effective intervention requires acknowledging the "causes of causes" — structural violence that generated parental weathering and persisting conditions sustaining transmission (Krieger, 2005).

Third, evaluating post-conflict health equity requires a multigenerational perspective rather than a cross-sectional assessment (Ataguba et al., 2015). Policymakers should track not only population health metrics but also intergenerational health gradients to assess whether biological legacies of historical injustices are being dismantled or perpetuated under new political arrangements.

### 4.7 Limitations

Several limitations warrant consideration. First, this study measured hypertension at single points in adulthood, missing blood pressure trajectories. Limited age overlap between cohorts means age and cohort effects cannot be fully disentangled. If parental weathering primarily affects offspring cardiovascular aging rates rather than baseline levels, our cross-sectional assessment might miss accelerated deterioration manifesting later (Mayosi et al., 2009; Pillay-van Wyk et al., 2016).

Second, selective migration could bias estimates if families most affected by apartheid were most likely to migrate or refuse assessments. However, NIDS's tracking protocols minimize attrition (Southern Africa Labour and Development Research Unit (SALDRU), 2018).

Third, the focus on hypertension captures only one manifestation of weathering (Bradshaw et al., 2006; Mayosi et al., 2009). Future research should examine whether cohort-invariant transmission extends to diabetes, obesity, and mental health.

Finally, the socioeconomic controls may not capture all dimensions of structural disadvantage. Residential quality, neighborhood safety, food environment quality, and wealth trajectories represent additional pathways through which structural conditions could sustain transmission (World Bank, 2022; Harris et al., 2011; Southern Africa Labour and Development Research Unit (SALDRU), 2018). The minimal 7% attenuation suggests these unmeasured factors explain limited variance, but more granular measures would strengthen mechanistic inference.

## 5 Conclusion

In post-apartheid South Africa, political liberation dismantled apartheid's legal architecture but failed to interrupt intergenerational cardiovascular transmission. Parent-offspring hypertension transmission operates with statistically invariant strength across birth cohorts, suggesting that mechanisms generating transmission — biological embedding, structural persistence, or their mutually reinforcing interaction — prove resilient to macro-political change.

This persistence reveals the stark reality of political liberation without health liberation. Democratic reforms, while introducing meaningful social policies, proved insufficient to break intergenerational health chains forged under structural violence (Leibbrandt et al., 2010; Southern Africa Labour and Development Research Unit (SALDRU), 2018). Whether this reflects biological damage irreversibly encoded before liberation (Barker, 1995; Gluckman et al., 2008; Kim et al., 2023), persistent structural disadvantage despite reforms (Chatterjee et al., 2022; Turok, 2014), or both operating simultaneously (Kim et al., 2023; Krieger, 2005), the policy implication remains clear: interrupting intergenerational transmission requires sustained, multi-generational commitment to both material redistribution and interventions targeting biological pathways.

These findings provide sobering evidence that breaking intergenerational chains of health inequality requires more than political transformation. Effective intervention demands sustained commitment to structural transformation — neighborhood reinvestment, healthcare strengthening, wealth redistribution, educational equity — alongside programs explicitly designed to interrupt biological transmission through maternal health services, early childhood nutrition, and stress reduction for weathered parents. Without such comprehensive efforts, apartheid's cardiovascular legacy will continue rippling across generations, binding present health to past violence long after formal liberation.

## Ethical statement

The National Income Dynamics Study received ethical approval from the University of Cape Town Commerce Faculty Ethics Committee. All NIDS participants provided informed consent. This study involves secondary analysis of publicly available, de-identified data that does not require additional ethical review.

## Declaration of interest statement

The author declares no competing interests.

## Data Availability

Data will be made available on request.
